# Free Medical Schools

**Published:** 1850-04

**Authors:** 


### EDITORIAL DEPARTMENT.

*Free Medical Schools.*—The project of free medical schools has lately been propounded as a new measure of medical reform. It has been advocated by Prof. Stevens, of the city of New York, in an eloquent address, delivered before the Medical Society of this State, and the Legislature, in February, 1849; and the subject was brought up at the meeting of the State Society, in Feb'y, 1850, and deferred for consideration at a future period. Dr. N. H. Davis, of Chicago, Ill., in an introductory address, delivered at the commencement of the Lectures in the Rush Medical College, 1849, espouses this as the grand measure of Educational progress. The plan has been adopted as the basis of the Medical department of the University of Michigan, which, we learn, is now organized, and is to go into operation during the next autumn. In this Institution, however, the sessions of instruction embrace the entire year, so that it will not afford an exemplification of the practical working of the system, which will be fairly applicable to schools holding sessions for a portion of the year only.

As it is not improbable that the new project may be submitted for the recommendation of the American Medical Association, and applications made to State Legislatures for its adoption, it is desirable that attention be directed to the subject, and that its merits and demerits be candidly considered, with a view to the formation of deliberate opinions, before members of the profession lend their influence either for, or against a measure, which certainly possesses considerable importance, inasmuch as, if adopted, it must lead to results, in no small degree favorable, or unfavorable, to the interests of Medical Education and science. We do not purpose, at this time, to enter into a full discussion of the subject, but we will suggest, for the reflection of our readers, some of the considerations which appear to render the proposed change objectionable and impolitic. We shall state the several points without amplification, since, as just stated, it is not our design to undertake an elaborate argument.

It may be remarked, in the first place, that a project involving so considerable an alteration in the organization of medical schools, should not be undertaken, or even advocated precipitantly. The profession should be well convinced that it will prove a real improvement, before they consent to unite in exerting their influence to secure its adoption. One reason why this consideration has peculiar force, is derived from the fact that, if the project be ever carried into effect, it must be done by legislative action on the part of State governments. The legislatures of the different States must be induced to commit themselves to this method of providing for medical education. Now, it has hitherto not been found an easy task to persuade legislatures to do much in behalf of the interests of the medical profession, or medical science. Assuming, for the moment, that they might be induced to listen to this new proposition, and give the scheme a fair experimental trial, it would behoove us to be well assured, beforehand, that the result would be satisfactory to all parties, else, future plans, emanating from the medical profession, would be likely to receive inadequate consideration. If the medical profession were to unite cordially on this project, and with great unanimity agree that it is very desirable it should be carried into effect, this would be, in itself, a striking fact; and perhaps the singularity and surprising character of such a spectacle might have no small influence in the attainment of the end. But were the end to be attained, and disappointment, as respects the expected advantages, to follow, nothing could be more unfortunate in view of any subsequent application for legislative co-operation.

Whatever confidence in the ability of the profession to decide on its own best interests might before have been felt, would, it is obvious, be greatly diminished by such a failure. The propriety of extreme caution, in yielding assent to this, or any other proposed measure of reform, the execution of which requires that it become an element of State policy, is too apparent to need further comment. We proceed, then, to an enumeration of some of the reasons which suggest themselves as militating against the establishment of free medical schools.

The First objection is, that the project is utterly impracticable in its accomplishment. This objection, if it be a valid one, is, of course, sufficient. To waste our combined energies in efforts which we can never hope will prove successful, is certainly worse than useless—it would be the extreme of folly. The probabilities of success should, therefore, be fairly weighed at the outset, and even before pains are taken to inquire into the intrinsic merits of the plan, provided it were feasible.

To render medical institutions free, they must be endowed sufficiently to afford adequate salaries to teachers, and for the various expenses incidental to medical instruction. To afford a decent compensation for the labor of a competent faculty, and to provide every thing necessary for carrying on a medical school properly and effectively, a considerable annual sum would be required. We will suppose that in this State our legislature should undertake to sustain three medical schools. At the lowest estimation, the annual sum required for their maintenance, collectively, would exceed thirty thousand dollars, exclusive of any appropriations for buildings, or for other extraordinary objects, such as providing a museum. This sum must be either appropriated annually, or an investment set aside, which would yield a corresponding amount by way of annual interest or revenue. Now we ask any one at all acquainted with the tone of public sentiment, or the temper of legislative bodies, on matters relating to the medical profession, whether there is even a possibility that an appropriation for such a pecuniary grant would be seriously entertained. But admit for arguments' sake, that an act were obtained by which the legislature made provision for the support of free medical schools for two years—the period fixed by the constitution for a prospective appropriation of money—would there be any security for the perpetuity of this policy? We may judge of this by the degree and kind of opposition which the medical profession now meets with from a not inconsiderable portion of the community. Would the Thomsonians, the Eclectics, the Homœopathists, and others, be satisfied to allow medical schools to bask in the sunshine of State liberality? That these enemies of the profession, although widely separated by incompatible doctrines, would combine, to a man, to effect the repeal of such a law, is much more certain than that the medical profession would unite to sustain it; and the consequence would be that the law would be repealed. We have seen what was the effect of making a discrimination in favor of the regular profession, in so far as to provide that the fees of the latter alone, for medical services, could be collected by legal processes. The legislature was importuned by petitions and remonstrances until this protective policy was abandoned, and no distinction made between the regular and irregular practitioner, as respects remuneration for professional services. We may infer from this what would be the opposition to a measure which not only committed the State more fully than ever in behalf of legitimate medicine, but which involves the responsibility of educating, gratuitously, its members. However devoutly to be wished were such a consummation, it is in vain to expect it in our day and generation.

We might call up various other obstacles. For examples: difficulties arising from the necessity of determining how many free schools should be supported, and where they should be situated; of deciding between the rival claims of different institutions in the same city; of annulling chartered rights, &c. All these points will doubtless occur to the mind of the reader.

A *second* objection to free schools applies to the policy of having a fixed salary for teachers, and rendering them responsible alone to government for the faithful discharge of their duties.

A stated compensation would tend to remove an incentive to exertion on the part of a teacher, which it were better should be left undisturbed. It may be said that conscientiousness, a proper sense of responsibility, and a due regard for character and reputation, furnish abundant security for fidelity, without need of the stimulus of filthy lucre. This may be plausable enough as an abstract proposition, but does it accord with our every day observation and experience. The consciousness that pecuniary reward for services is to be measured, in part, at least, by the degree of effort to render the services truly valuable, possesses an influence which the shrewd observer cannot fail to appreciate in others, and which a candid self-examination will reveal in the experience of almost every one, whatever may be the nature of the duties for which a compensation is expected. It is true, that the organization of other educational institutions is not always based on this maxim of human life. We have only to say, in answer to this, that we believe it would be better if it were otherwise. We do not doubt that it would be an improvement in all our academical institutions, if the emoluments of those engaged in instruction were, to a greater or less extent, made dependent on the success of the institutions as denoted by the number of pupils which they respectively attract.

Moreover, in medical schools, it seems to us desirable that teachers should not be too independent, in their official positions, of their classes. They should be, in a measure, responsibie to them. The present order of things, in this respect, although affording a license which admits of abuse, nevertheless is conducive to a wholesome discipline. The position of a medical teacher, in this country, involves a perpetual *concours*. If he fail to satisfy the wishes of those who are obliged to listen to his instructions, his tenure of office is more precarious than if he and his associates were less dependent on the number of pupils which their combined reputation brings together.

We might illustrate these positions by examples. We could cite medical institutions where salaries are paid by government, and verify the correctness of our remarks by comparing them with others in which no such collateral aid is received. But this we prefer to leave for the inquiries of our readers.

A *third* objection is, that appointments for the office of teacher would be less likely to be based on capacity and acquirement than under the present system. On a superficial view of the subject, it may seem that the reverse of this statement would be nearer the truth, but, on due reflection, we are persuaded the correctness of the assertion will be apparent. If State governments provided for the maintenance of medical schools, they would, of course, reserve the right, directly or indirectly, to control the appointments. Now this being the case, is there not every reason to suppose that they would very soon, if not at once, become mere political appointments? This is the case with the medical appointments now made by state, county, and municipal authorities: what security could there be that these new offices would not share the same fate? Every change of administration, then, would involve a change in the Faculty of medical colleges, the new *regime* conforming, in political complexion, to the party in power. It is needless to discuss the policy of such a state of things in so far as the interests of medical education are concerned. The question simply is, whether medical institutions would be better off, if places therein were to be obtained through political intrigues, claimed as rewards for party services, or filled by incumbents whose qualifications consist in their being relatives of persons in high positions, and in their inability to sustain themselves as private practitioners! We leave this question to be answered by the reader.

A *Fourth* objection to free schools is, that they would tend to multiply practitioners beyond the wants of society, and the interests of the profession. It is already a subject of complaint with many, that the ranks of the medical profession are too full—that the competition arising from over crowding, leads to serious evils incident to a depreciation of the dignity and value of professional services. If there be any ground for such complaints under the present system of medical education, surely matters would not be improved by opening wider the doors, and inviting all to enter without money or price! The expense of obtaining a medical education, while it is not sufficiently great to prohibit any one from entering the profession, however limited may be his resources, provided he has sufficient energy and talent to succeed in the profession, after the obstacles opposing his progress are met and overcome, nevertheless is, in some measure, a tarif, which serves the purpose of protection against an overplus alike injurious to the interests of the profession and of society. Moreover, one effect of gratuitous instruction would be, that many persons poorly qualified, by nature or previous education, to become respectable, useful members of the profession, would be among those seeking admission, who are now diverted into pursuits better adapted to their capacities, in consequence of the *impost* involved in the present system.

The *Fifth*, and last of the objections which we shall at present adduce, would be applicable if any State or States should provide for free medical schools, while other States could not be persuaded to act in concert—in other words, the objection would hold good so long as the policy of gratuitous instruction was not universal throughout the United States. The advocates of the project would doubtless say, in reply to the fourth objection, that it is designed to increase the period of study, and requisites for graduation, while pecuniary restrictions are removed, and that these new provisions would operate as a measure of protection against too great increase of members, far more efficiently than an impost of money. It is doubtful whether this reasoning be correct, but we will not now stop to question it. Assume it to be true, and suppose the *Empire State* to adopt the policy of free medical schools, with restrictive provisions as to term of study, preliminary acquirements, etc., to suit the views of the medical reformers of the day. It would be easy for students to avail themselves of the gratuitous instruction offered in this State, and graduate at the medical institutions of other States, where the requisitions for graduation were fewer. This would certainly be done, if it be true, as stated by Prof. Davis, in an article, some time since, on the subject of medical schools, that students will always give preference to those institutions where degrees are obtained on the easiest terms. We do not concur with Prof. D. in this opinion, but it is altogether probable, that if the requirements for graduation in this State were deemed unduly onerous, under the supposed arrangement, they would decline to comply with them, while other institutions having equal character and advantages, showed greater liberality in this respect. It will be said, perhaps, that this objection might be obviated, in so far as this State is concerned, by passing a law prohibiting physicians from practising, with diplomas from institutions obtained in other States. It cannot, however, be reasonably expected, by any one, that any State would pass such an act, or, if passed, that it would be permitted to remain unrepealed. The policy pursued by almost every State in the Union toward the medical profession is diametrically opposed to such a course.

In closing this article we will say a word or two respecting *medical reforms* in general. The end aimed at in all the measures of reform proposed, is the same—to wit: to elevate the character of the medical profession. In this sense we claim to be an humble advocate for reform; not that the profession is degraded—not that it is receding, instead of advancing in character and usefulness, commensurately with the constant progress of medical science—but because we believe it is capable of becoming more and more elevated, and that there are no limits to its advancement. No one, certainly, will object to reformation in this sense, although, we confess, the word is not exactly to our taste. The question, then, is, how shall members of the profession individually contribute to elevate the profession? We answer, first and chiefly, by elevating themselves—increasing their own acquirements, and keeping pace with the progress of medical knowledge; cultivating respect for the profession, and love of the science; respecting, more and more, in their relations with their brethren, the law of brotherly love and kindness, which will render a code of medical ethics quite superfluous; and in all other particulars adopting, as a rule of action, *self-elevation.* In our opinion the reforming progress would be accelerated by more attention to the subject in its personal applications. There is much more said on the subject, intended to apply to the profession in general, than would be said, if a keener sense of individual responsibility, in the matter, were always felt. Let any person resolve to employ his best energies to keep himself tolerably conversant with the new developments that are constantly transpiring in the progress of medical knowledge, and, if we mistake not, he will not only find himself at a loss for time to reflect much on the deficiencies of his neighbors, and the ignorance of the profession at large, but distrusting his own ability to accomplish the task before him, he will have but little inclination to study how far others fall short of the mark at which he aims. It is an obvious truth, that if every member of the medical profession were to be fully imbued with such a spirit of self-improvement, the progressive elevation of the profession would be completely secured, and with this observation we leave the subject.

